# Enhanced Oral Bioavailability of β-Caryophyllene in Healthy Subjects Using the VESIsorb^®^ Formulation Technology, a Novel Self-Emulsifying Drug Delivery System (SEDDS)

**DOI:** 10.3390/molecules27092860

**Published:** 2022-04-30

**Authors:** Yvonne Mödinger, Katharina Knaub, Tanita Dharsono, Roland Wacker, Remo Meyrat, M. Hunter Land, Anthony L. Petraglia, Christiane Schön

**Affiliations:** 1 BioTeSys GmbH, Schelztorstraße 54-56, 73728 Esslingen, Germany; y.moedinger@biotesys.de (Y.M.); k.knaub@biotesys.de (K.K.); t.dharsono@biotesys.de (T.D.); r.wacker@biotesys.de (R.W.); 2 Interlabor Belp AG, Aemmenmattstrasse. 16, 3123 Belp, Switzerland; remo.meyrat@interlabor.ch; 3 Osage Research and Development, 734 Cutter Ct., Kure Beach, NC 28449, USA; michaelhunterland@gmail.com; 4 Division of Neurosurgery, Rochester Regional Health, Unity Hospital, 1555 Long Pond Rd., Rochester, NY 14626, USA; anthony.petraglia@rochesterregional.org

**Keywords:** beta-caryophyllene (BCP), bioavailability, hemp, *Cannabis sativa* L., human, oral drug delivery system, pharmacokinetic, SEDDS, VESIsorb^®^ formulation technology, endocannabinoid system, nutraceuticals

## Abstract

β-Caryophyllene (BCP), a common constituent of many spice and food plants, is gaining increased attention due to recent research identifying numerous potential health benefits. Due to limited oral bioavailability observed in preclinical models, the described benefits of BCP may be maximized by using a suitable delivery system. Additionally, human pharmacokinetics (PK) remain unknown. This study evaluates the relative oral bioavailability of BCP formulated in a self-emulsifying drug delivery system (SEDDS) based on VESIsorb^®^ formulation technology (BCP-SEDDS) compared to BCP neat oil. Hence, a randomized, double-blind, cross-over design, single oral dose study (100 mg BCP) in 24 healthy subjects (12 men/12 women) was performed under fasting conditions. Pharmacokinetic parameters were analyzed from individual concentration-time curves. The data show that BCP-SEDDS resulted in a 2.2/2.0-fold increase in AUC_0–12h_/AUC_0–24h_ and a 3.6-fold increase in C_max_ compared to BCP neat oil. Moreover, BCP was absorbed faster from BCP-SEDDS (T_max_: 1.43 h) compared to BCP neat oil (T_max_: 3.07 h). Gender analysis revealed that there is no significant difference between men and women for both the investigated formulations and all investigated PK endpoints. In conclusion, BCP-SEDDS offers a well-tolerated and effective oral delivery system to significantly enhance the oral bioavailability of BCP in humans.

## 1. Introduction

β-caryophyllene (BCP) is a natural bicyclic sesquiterpene and a common constituent of the essential oils of numerous spice and food plants, such as clove, black pepper, melissa, rosemary, basil, lavender, oregano, hops, and hemp (*Cannabis sativa* L). BCP, although chemically unrelated to classic phytocannabinoids, is thought to interact with the endocannabinoid system by binding selectively to cannabinoid receptors type 2 (CB2). Unlike delta-9-tetrahydrocannabinol, BCP lacks appreciable functional activity at cannabinoid receptor type 1 (CB1), thus devoid of intoxicating effects [1,2]. Furthermore, considerable data exist describing the various actions of BCP and its modulation of targets unrelated to the endocannabinoid system. Some of these additional targets include peroxisome proliferator-activated receptors-α (PPAR-α) and PPAR-γ [3].

Several biological activities of BCP have been reported in experimental studies, including anti-microbial [4], anti-carcinogenic, and analgesic effects [5,6,7,8,9]. Furthermore, anti-inflammatory [10], immune-modulatory [11], antioxidant [12,13,14,15], anxiolytic and antidepressive [16,17,18], as well as hepato- [19] and neuroprotective activities [20,21] are discussed for BCP.

Studies in animals showed that BCP exhibits poor oral bioavailability and, hence, its health benefits in the nutraceutical and pharmaceutical fields may not be fully exploited. The low solubility of BCP in aqueous media such as biological fluids, its high volatility, and its high sensitivity to oxidation when exposed to light or oxygen are thought to be reasons for the poor bioavailability [22,23,24,25]. Furthermore, the uptake of BCP is subject to a high inter-individual variability [1,24]. The use of drug delivery systems such as solid lipid nanoparticles, inclusion complexes with cyclodextrins, nanoemulsions, liposomes, and coprecipitated microparticles has been proposed to overcome these limitations [23,26,27].

Self-emulsifying drug delivery systems (SEDDS) represent another approach to improving the oral bioavailability of poorly water-soluble substances that have gained increased interest. SEDDS are isotropic mixtures of drugs, oils, emulsifiers, and optionally hydrophilic solvents. They spontaneously emulsify upon contact with an aqueous phase, such as gastric or intestinal fluids, and thereby keep the co-administered lipophilic active solubilized within their oil droplets. The drug-containing oil droplets are diffused through the aqueous intestinal lumen and the unstirred water layer to the enterocyte surface. At the enterocyte surface, single molecules of the active ingredient partition from the oil droplet to the enterocyte membrane (flip-flop) and then further into the blood circulation or to the lymphatic system. By varying the composition of SEDDS, custom-made solutions can be developed that fit both the compound of interest and the target [28,29,30,31,32]. Most SEDDS are liquid and are administered either in hard or soft gelatin capsules. However, liquid SEDDS can also be converted to solid SEDDS, enabling the manufacturing of other solid dosage forms such as tablets [33].

VESIsorb^®^, a SEDDS platform technology developed by Vesifact AG, Baar, Switzerland, has been shown to increase the oral bioavailability of poorly water-soluble molecules such as coenzyme Q10 (ubiquinone) [34] and the phytocannabinoid cannabidiol (CBD) [35] in human pharmacokinetic trials. The applied SEDDS were tailor-made with respect to composition and droplet size for maximum bioavailability enhancement. For example, a single oral dose of CBD in VESIsorb^®^ led to a 4.4-fold higher C_max_ and a 2.85-/1.70-fold higher AUC_0–8h_/AUC_0–24h_ compared to the reference formulation of CBD in medium-chain triglycerides after consumption by healthy human subjects [35].

To our knowledge, no human oral bioavailability data has been published to date. Thus, the objective of the present study was to evaluate whether VESIsorb^®^-based SEDDS can improve the relative oral BCP bioavailability compared to neat oil. To address this, a randomized, double-blind, cross-over design clinical trial under fasting conditions and a controlled diet was performed. The BCP concentration-time profile, the area under the curve (AUC), maximum plasma concentration (C_max_), and time to maximum blood concentration (T_max_) were determined in 24 healthy subjects (12 men and 12 women) receiving a single-dose (standardized to 100 mg BCP), either as a SEDDS formulation (BCP-SEDDS) or as neat oil (BCP neat oil, reference product).

Overall, results showed that BCP is absorbed after oral administration of either BCP neat oil or BCP-SEDDS and that BCP-SEDDS significantly improved all determined the following pharmacokinetic parameters: increased BCP plasma concentration (C_max_), enhanced bioavailability (AUC), and faster absorption (T_max_) as compared to BCP neat oil.

## 2. Results

### 2.1. Subject Characteristics

The investigated study population (*n* = 24) was healthy, non-smoking, and on average 27.9 years old (95% confidence interval (CI): 25.2–30.6) with a BMI of 23.0 kg/m^2^ (95% CI: 22.2–23.8). Table 1 specifies the demographic data of all subjects as well as separated by gender. The groups of women and men were comparable in terms of age, BMI, and total cholesterol level. Men showed significantly higher systolic (*p* = 0.0068) and by trend diastolic (*p* = 0.0511) blood pressure values in comparison to women. Anyhow, vital signs and blood routine parameters were within the normal range.

### 2.2. BCP Plasma Concentration Time Profile

**Figure 1:**
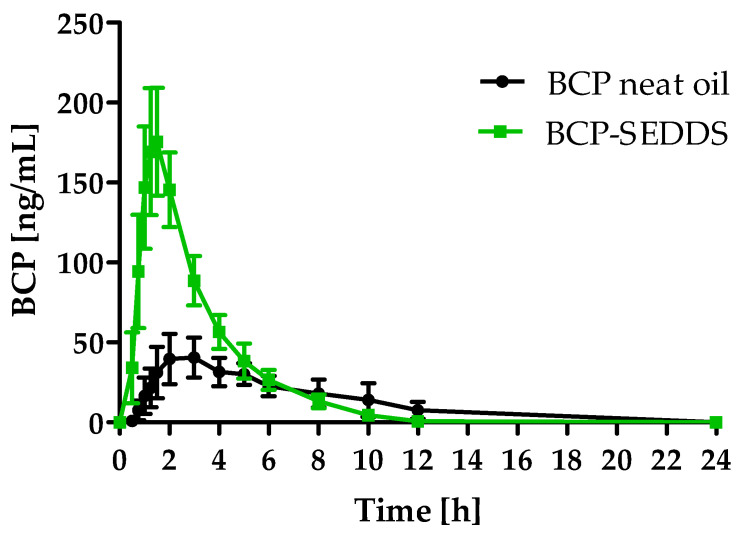
Beta-caryophyllene (BCP) plasma concentration time profile after a single oral dose of 100 mg BCP administered as self-emulsifying drug delivery system (BCP-SEDDS, green) and BCP neat oil (reference, black) depicted as summary curves of mean values at single time points (mean ± 95% CI) for all subjects. *n* = 24.

After the application of the study products, BCP plasma concentration was monitored over the course of 24 h. There was a significant increase in BCP plasma concentration over time in both formulations (*p* < 0.0001). For BCP-SEDDS, the concentration significantly increased starting at 0.75 h post-dosing, compared to baseline levels. For BCP neat oil, the concentration-time curve was at a much lower level and increased later at 1.25 h post-dosing, in comparison to baseline (Figure 1). After 10 h (BCP-SEDDS) and 12 h (BCP neat oil), most of the BCP was metabolized and/or eliminated, reaching nearly baseline levels (Figure 1).

### 2.3. Pharmacokinetic Parameters of BCP

Pharmacokinetic parameters were analyzed from individual concentration-time curves. The AUC of BCP-SEDDS after a single oral dose (100 mg BCP) was significantly higher for both AUC_0–12h_ and AUC_0–24h_. AUC_0–12h_ was 549.5 and 260.7 ng/mL × h (*p* < 0.0001) and AUC_0–24h_ was 553.4 and 305.9 ng/mL × h (*p* < 0.0001) for BCP-SEDDS and BCP neat oil, respectively (Table 2, all subjects). An inter-individual coefficient of variation (CV) of 60.1% (BCP neat oil) and 42.1% (BCP-SEDDS) was observed for AUC_0–24h_.

The BCP maximum plasma concentration (C_max_) was also significantly higher after BCP-SEDDS compared to BCP in neat oil. C_max_ values were 204.6 ng/mL and 58.22 ng/mL for BCP-SEDDS and BCP neat oil, respectively (Table 2, all subjects). The C_max_ data thus confirm the superiority of BCP-SEDDS over BCP in neat oil (*p* < 0.0001). For this endpoint, the CV was 53.3% (BCP neat oil) and 43.4% (BCP-SEDDS).

Additionally, absorption of BCP from BCP-SEDDS was significantly faster compared to BCP in neat oil (*p* = 0.0003). T_max_ was 1.43 h for BCP-SEDDS and 3.07 h for BCP in neat oil (Table 2, all subjects).

The significant improvement in the oral bioavailability by the BCP-SEDDS formulation in comparison to BCP neat oil was also confirmed in men and women separately for all pharmacokinetic parameters (Table 2, men and women).

### 2.4. Effects of Gender on BCP Pharmacokinetics

**Figure 2:**
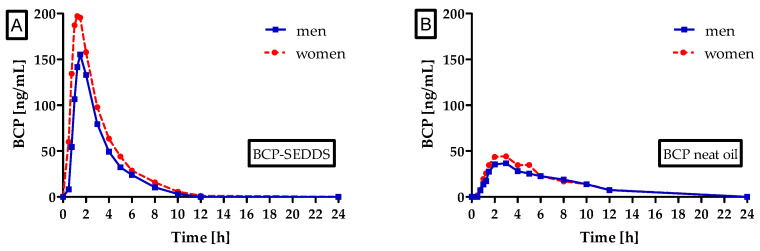
Beta-caryophyllene (BCP) plasma concentration time profile after a single oral dose of 100 mg BCP administered as self-emulsifying drug delivery system (BCP-SEDDS) (**A**) and BCP neat oil (**B**) depicted as summary curves of mean values at single time points for men (blue, solid line) and women (red, dotted line).

Next to the investigation of differences in bioavailability of BCP from BCP-SEDDS and neat oil, the uptake characteristics within the formulations were compared between women and men. Concentration-time curves summarizing men and women separately are shown in Figure 2. There is no significant difference between men and women for both formulations and all pharmacokinetic parameters. However, for BCP-SEDDS, there is a trend for increased bioavailability in women in comparison to men for both AUC_0–24h_ (*p* = 0.0520) and AUC_0–12h_ (*p* = 0.0525). Albeit higher concentrations of C_max_ were measured in women (233.0 ng/mL) in comparison to men (176.3 ng/mL), the significance level was missed (*p* = 0.1162). No gender differences were observed for T_max_.

### 2.5. Bioequivalence Assessment

Overall, a bioequivalence assessment of 90% CI for the ratio of geometric means further confirmed the superiority of BCP-SEDDS compared to the reference (BCP neat oil) for all evaluated pharmacokinetic parameters. The ratio of geometric means resulted in an AUC of a factor of 2.0–2.4 and a C_max_ of a factor of 3.4–4.0 in favor of BCP-SEDDS (Table 3). This underlines the biological relevance of the observed differences in bioavailability as the lower bound of the 90% CI clearly exceeds the bioequivalence criterion of 1.25 for all investigated endpoints as well as subgroups of women and men (Table 3).

### 2.6. Safety Assessment

Both study products were well-tolerated by all subjects (100%). No treatment-related adverse events were reported. Recorded adverse events within 24 h post-dosing included headaches (4 subjects) and dizziness after blood withdrawal (2 subjects). One subject took a pain reliever (Ibuprofen) because of a headache after the 12 h time point after having left the study site. There was no serious adverse event, and none of the adverse events were judged as severe. Recorded vital signs and blood routine parameters of all subjects were in accordance with reference values and were not clinically relevant. The results of this study do not raise any safety concerns.

## 3. Discussion

β-caryophyllene (BCP) is emerging as an interesting active ingredient because of its potential for health benefits as a result of the multi-model activity. Preclinically, BCP selectively binds to the CB2 receptor but lacks functional activity at the CB1. Unlike CB1 receptor agonists, BCP is devoid of intoxicating effects. PPAR α/γ activity, known to play a major regulatory role in energy homeostasis and metabolic function, has also been highlighted as an important mechanism of action. A number of other targets have been identified in vitro, although their potential physiological implications are currently theoretical. Published benefits contributed to BCP include improvement in atherosclerosis, osteoporosis, colitis, osteoarthritis, inflammatory conditions, diabetes, anxiety, depression, and reduction in pain. Notably, BCP exhibits no abuse potential itself while showing promising results in the treatment of addiction-like behaviors and substance abuse disorders involving e.g., cocaine, ethanol, nicotine, or opioids via a PPAR α/γ-dependent pathway [36,37]. Therefore, BCP might enable the long-term treatment of chronic pathologies without the development of addiction-like behavior or dependence. Additionally, due to its anxiolytic effects, BCP has been proposed as a possible substitute for benzodiazepines and selective serotonin reuptake inhibitors (SSRI). Both substances are known to have severe side effects [38]. In contrast, BCP per se shows an excellent safety profile. It is approved by both the United States Food and Drug Administration (FDA) and the European Food Safety Authority (EFSA) as a food additive, taste enhancer, and flavoring agent, and is listed as a Generally Recognized as Safe (GRAS) III Substance [39]. Furthermore, a No Observed Adverse Effect Level (NOAEL) for BCP of 222 mg/kg body weight per day was stated by the EFSA scientific opinion [40], based on a 90-day GLP-compliant repeat dose toxicity study in rats, where the safety of BCP was assessed at three different dietary concentrations, showing no deaths or other signs of clinical toxicity [41]. In addition, acute and repeated-dose toxicity studies in *Swiss* mice with 300 and 2000 mg BCP/kg were performed according to the Organization for Economic Cooperation and Development (OECD) guidelines 423 and 407, respectively. No adverse clinical signs or impact on metabolic effects were observed in the single-dose and repeated-dose toxicity studies [42]. A subchronic toxicity study in rats with dosages up to 700 mg/kg/d for 90 days also underlines the good safety profile [43]. The good tolerability was also confirmed in the present clinical study with a single dose of 100 mg BCP.

Based on animal data, Santos et al. deduced a low oral bioavailability in humans and thus suggested that formulation approaches are a hard requirement for successful and sufficient delivery of BCP to humans [23]. To overcome the bioavailability issues and to maximize the benefits of BCP, a novel self-emulsifying drug delivery system (SEDDS) based on the VESIsorb^®^ formulation technology was tailor-made for BCP (BCP-SEDDS).

The herein presented data show that BCP-SEDDS provided improved bioavailability over BCP neat oil. A single oral dose of BCP-SEDDS resulted in a 2.2/2.0-fold increase in AUC_0–12h_/AUC_0–24h_ and a 3.6-fold increase in C_max_ compared to BCP neat oil (based on geometric means, all subjects). In addition, T_max_ was reached faster (1.43 h BCP-SEDDS vs. 3.07 h BCP neat oil).

Gender analysis revealed that there was no significant difference between men and women for both formulations and all pharmacokinetic parameters. For BCP-SEDDS, a trend towards increased bioavailability in women compared to men was observed for AUC but not for C_max_. It cannot be excluded, however, that this finding only reflects inter-individual variability, and it should therefore be confirmed in future studies.

A sound comparison of the herein measured pharmacokinetic endpoints with literature data is not possible as, to our knowledge, there are no other human bioavailability studies on BCP. In non-human studies, on the other hand, different delivery systems for BCP have been investigated, among them polymeric nanoparticles, inclusion complexes with cyclodextrins, and nanoemulsions, as reviewed by Santos et al. [23]. A study conducted in rats by Liu et al. showed that drug delivery systems such as cyclodextrins can indeed improve bioavailability compared to neat oil [25].

The mechanisms by which SEDDS increase the oral bioavailability of BCP are not fully understood yet. The following mechanisms can be envisioned: First and foremost, the solubilization of BCP in the aqueous environment of the gastrointestinal (GI) tract is thought to be the main mechanism. Second, BCP has been shown to be a substrate of efflux transporters [44,45]. Thus, it is possible that components of the SEDDS formulation can inhibit such transporters, thereby reducing the BCP transport from the enterocyte back to the lumen of the GI. Third, it can be envisioned that components of the SEDDS formulation are inhibiting metabolizing enzymes. However, this is unlikely since the elimination of BCP from the circulation was comparable to or even faster with BCP-SEDDS compared to BCP neat oil, see Figure 2. The fourth possible mechanism of action, a reduction of liver first-pass metabolism by directing the absorption of BCP towards the lymphatic pathway, is likewise unlikely since the shorter T_max_ values achieved with BCP-SEDDS compared to BCP neat oil directly contradict the concept of lymphatic absorption.

The presented data further show that at the 24 h measurement time point, none of the subjects showed detectable BCP concentrations, and even after 12 h, BCP concentrations were below the limit of quantification in most subjects after both tested formulations, suggesting a rapid metabolism of BCP in the body. Future investigations should focus on the processes of BCP first-pass metabolism and its metabolism in the liver, as well as on its excretion. Moreover, additional data is needed regarding desired plasma levels or thresholds of BCP concentrations and the correlation of these values with BCP efficacy. Of note, BCP efficacy was not investigated in the presented study.

To control for confounding factors such as additionally consumed food prior to or during the trial, this study was conducted under fasting conditions and with a study diet devoid of BCP-containing foods. Moreover, the cross-over study design accounts for the high inter-individual BCP bioavailability, described e.g., in laboratory animals (coefficient of variation for AUC_0–12h_ = 44.9%) [25]. Inter-individual variability of AUC_0–24h_ values was 42.1% (BCP-SEDDS) and 60.1% (BCP neat oil) in the current study, which is comparable to published data. Given the fact that laboratory animals represent a highly standardized model, it was expected that the inter-individual variability would be higher. Hence, the observed low inter-individual variability of 42.1% for BCP-SEDDS (AUC) is unexpected and further demonstrates the utility of the SEDDS technology for the delivery of BCP.

Limitations of the presented study comprise the inability to determine intra-individual variability of BCP bioavailability from the data, as study products were only provided once to subjects. In addition, the study data does not show how BCP bioavailability is affected by food intake and under-fed conditions. Compare the pharmacokinetics of BCP under fasting and fed conditions, thus representing a topic to be investigated in the future. Furthermore, future evaluations of the distribution, metabolism, and excretion of BCP would be highly valuable and helpful in optimizing the use of BCP in humans and exploiting its numerous beneficial effects.

## 4. Materials and Methods

### 4.1. Study Subjects

**Figure 3:**
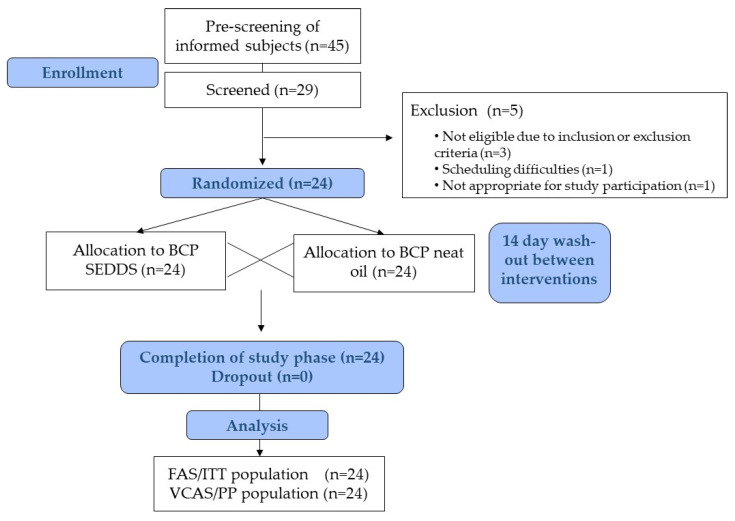
Study flow chart and subject disposition.

Figure 3 depicts the screening and enrolment process, during which 45 subjects were pre-screened for study eligibility, whereof 29 subjects were invited for screening visits. According to inclusion criteria, subjects had to be non-smokers, aged between 18 and 50 years, and have a body mass index (BMI) of 19–30 kg/m^2^. In addition, subjects were to be in good physical and mental health condition as established by their medical history, and by a physical examination including electrocardiogram, vital signs as well as results of biochemistry and hematology. Main study exclusion criteria were a relevant history or presence of any medical disorder, potentially interfering with this study (e.g., malabsorption, chronic gastrointestinal diseases, cardiovascular disease event within last 3 months), drug-, alcohol- and medication abuse or the regular intake of drugs or supplements possibly interfering with study results, as well as use of foods, spices and oils containing BCP (mainly black pepper, melissa, rosemary, oregano, cinnamon, thyme, and hemp) at least 48 h prior to the kinetic days to exclude possible interactions. Medications for treatment of chronic diseases not affecting the metabolism of the study products were permitted after individual judgment by the investigator regarding potential study interference. Any used concomitant chronic medication and medication for the treatment of adverse events were documented. Reasons for non-inclusion of 5 subjects were 1) exceeding the BMI cutoff 2) acute intake of antimycotics against fungal infection 3) cholesterol and triglyceride levels exceeding the normal range 4) schedule difficulties and 5) poorly accessible veins rendering the subject inappropriate for study participation. Finally, 24 subjects (12 men, 12 women) were included and all completed the study in its entirety (Figure 3).

### 4.2. Study Design

The clinical study was performed as a randomized, double-blind, monocentric, and controlled cross-over design at the study site of BioTeSys GmbH (Esslingen, Germany), comprising two in-clinic stays and a 14-day washout period in between. In total, 24 healthy subjects (50% of each gender) were randomized to receive a single oral dose of 100 mg BCP both as either SEDDS-BCP or BCP neat oil together with 250 mL of still water under fasting conditions (10 h overnight and 4 h post-dosing). Blood samples were collected at pre-dosing and 0.5, 0.75, 1, 1.25, 1.5, 2, 3, 4, 5, 6, 8, 10, 12, and 24 h after product administration. An indwelling catheter was placed in the vein for the consecutive blood samples for 12 h. During this time subjects stayed at study site. After 12 h subjects went home for sleeping and returned to study site the next morning for the 24 h blood sampling. All meals, including dinner on the evening prior to the kinetic days, were standardized until 24 h post-dosing, as was fluid intake during the intervention until 12 h post-dosing. Thereafter *ad libitum* but limited to water. Meals were served during kinetic days at 4 h (white bread with butter, boiled egg, and a glass of orange juice), 8 h (spaghetti carbonara), 10 h (apple and cookie), and 12 h (farmhouse bread with ham, carrot, strawberry yogurt) post administration of study products. Furthermore, subjects were advised to avoid alcohol 24 h, and BCP-containing oils and foods 48 h before the study intervention and to abstain from strenuous physical activity or endurance sports 24 h prior to the intervention. During the course of the study, subjects documented any adverse events and concomitant medication in diaries. The overall tolerability of the ingested study products was assessed at the end of each kinetic day.

The study was registered in the German Clinical Trials Register (DRKS00020461).

### 4.3. Intervention

BCP distilled from clove oil, formulated as SEDDS based on the proprietary VESIsorb^®^ technology (BCP-SEDDS) or as neat oil without any additional ingredient (BCP neat oil), were provided as single dose, each standardized to 100 mg BCP. BCP-SEDDS are composed of BCP, food emulsifiers, edible vegetable oils, and fatty acids. Characterization of the SEDDS formulation was carried out by determining the content of BCP and measuring the size of the droplets formed after dilution of BCP-SEDDS with purified water at 37 °C. The mean diameter of the droplets is from 40 to 50 nm (volume-weighted) as assessed by dynamic light scattering (Zetasizer Nano S, Malvern Instruments Limited, Worcestershire, UK). The formed droplets are very homogeneous exhibiting only one population as indicated by a very low polydispersity index of less than 0.100. Both formulations were filled into colored hard-shell capsules delivering 100 mg BCP per capsule yielding the final study products. The storage stability of both study products is given for at least two years at 25 °C as assessed by emulsification/droplet formation (BCP-SEDDS) and BCP content (BCP-SEDDS, BCP neat oil). Manufacturing of the BCP-SEDDS formulation and filling of either BCP-SEDDS or BCP neat oil formulation into capsules were carried out in compliance with GMP conditions. All excipients as well as the capsule shell met the current European food regulations. The investigational (BCP-SEDDS) and the reference (BCP neat oil) products were fully identical regarding color, size, odor, and secondary packaging to ensure double-blind conditions. Capsules were provided by Vesifact AG (Baar, Switzerland).

### 4.4. Sample Analysis

Blood samples for analysis of safety parameters were collected at screening and pre-dosing and 24 h post-dosing on both kinetic days. Safety parameters (differentiated hematogram and clinical laboratory including lipid status and liver enzymes) were analyzed at an accredited lab (Synlab Medizinisches Versorgungszentrum Leinfelden-Echterdingen, Germany) the same day. Venous blood, collected in EDTA monovettes (Sarstedt, Germany), was processed under light-protected conditions and centrifuged at 3000× *g* for 10 min at 4 °C. Processing time was below 60 min until freezing at −80 °C of plasma aliquots. GC-MS/MS operated in electron ionization (EI) multiple reaction monitoring (MRM) mode was used for determination of BCP concentration in EDTA treated plasma. Detailed GC-MS/MS conditions are provided in Supplementary Table S1 and Figure S1. In brief, plasma was thawed and briefly vortexed and 200 µL *n*-Hexan containing 400 ng/mL Humulene as internal standard was added to 400 µL of plasma sample, before vortexing and extraction on the shaker for approx. 30 min. After centrifugation and removal of the upper phase (160 µL), the extraction process was repeated. Both extracts were combined and injected (2 µL) into a Zebron^TM^ ZB-MR1ms column (Phenomenex^®^) for measurement with 1.0–1.2 mL/min flow rate. Quantification was performed against the calibration (external calibration with internal standard correction). Interassay precision was 6.2% and limit of quantification was 10 ng/mL.

### 4.5. Analysis Software and Statistical Analysis

Pharmacokinetic endpoints were determined from the individual plasma concentration-time curves. Area under the observed concentration-time curve above baseline after 12 h and 24 h (AUC_0–12h_ and AUC_0–24h_) was calculated by applying the trapezoidal rule with the *y*-axis defined by BCP plasma concentration and the *x*-axis defined by the sampling time points. Plasma concentrations below the lower limit of quantification at early and late time points were treated as zero. Curve progression was analyzed by Friedman test. AUC_0–12h_, AUC_0–24h_, and C_max_ were analyzed after log transformation using a linear mixed model taking into account sequence, period, and product. T_max_ was evaluated with a non-parametric test (Wilcoxon rank sum) taking into account sequence, period, and product. All statistical tests were performed two-sided and *p*-values < 0.05 were considered statistically significant. All 24 subjects were included in the analysis. Unless stated otherwise, data are presented as mean ± 95% CI. Statistical evaluation and graphs were generated using GraphPad Prism software (La Jolla, CA, USA) and IBM SPSS statistics 24 software (IBM, New York, NY, USA).

## 5. Conclusions

The objective of the current study was to evaluate, for the first time, the oral bioavailability of SEDDS-formulated BCP using the VESIsorb^®^ formulation technology (BCP-SEDDS) compared to BCP neat oil in healthy men and women under fasting conditions. Overall, the oral bioavailability of BCP was significantly higher, and BCP was absorbed significantly faster when administered as BCP-SEDDS compared to BCP neat oil. Moreover, no gender differences were observed for the investigated pharmacokinetic endpoints AUC, C_max_, and T_max_ with either BCP-SEDDS or BCP neat oil.

To conclude, BCP formulated as SEDDS offers a well-tolerable and effective delivery system to enhance the oral bioavailability of BCP in humans and may thus maximize the many health benefits offered by this molecule.

## Figures and Tables

**Table 1:** Demographic and baseline data.

Variable	All		Men *		Women *	
	Mean	(95% CI)	Mean	(95% CI)	Mean	(95% CI)
Age (years)	27.9	(25.2–30.6)	28.8	(24.6–33.1)	26.9	(22.9–31.0)
BMI (kg/m^2^)	23.0	(22.2–23.8)	23.4	(22.5–24.3)	22.6	(21.2–24.1)
Systolic BP (mmHg)	122.9	(116.9–128.9)	130.3	(122.3–138.4)	115.4	(107.9–122.9)
Diastolic BP (mmHg)	80.6	(75.7–85.6)	85.3	(77.2–93.3)	76.0	(70.3–81.7)
Hemoglobin (g/dL)	14.2	(13.7–14.7)	14.9	(14.1–15.7)	13.4	(13.0–13.9)
Total Cholesterol (mg/dL)	169.0	(157.0–181.0)	171.7	(150.7–192.6)	166.3	(151.0–181.7)

BMI: body mass index; BP: blood pressure; CI: confidence interval. * *n* = 12.

**Table 2:** Pharmacokinetic parameters of BCP after a single oral dose of 100 mg BCP administered as self-emulsifying drug delivery system (BCP-SEDDS) and as BCP neat oil (reference).

Subjects	Pharmacokinetic Parameters											
	C_max_ [ng/mL]			AUC_0–12h_ [ng/mL × h]			AUC_0–24h_ [ng/mL × h]			T_max_ [h]		
	Mean (95% CI)			Mean (95% CI)			Mean (95% CI)			Median (25th–75th Percentile)		
	BCP- SEDDS	BCP Neat Oil	*p*	BCP- SEDDS	BCP Neat Oil	*p*	BCP- SEDDS	BCP Neat Oil	*p*	BCP- SEDDS	BCP Neat Oil	*p*
All (*n* = 24)	204.6 (167.2–242.1)	58.22 (45.13–71.3)	<0.0001	549.5 (456.4–642.7)	260.7 (203.7–317.7)	<0.0001	553.4 (455.1–651.7)	305.9 (228.2–383.5)	<0.0001	1.5 (1.0–1.9)	3.0 (2.0–5.0)	0.0003
Men (*n* = 12)	176.3 (124.6–228.0)	58.19 (33.12–83.26)	0.0001	461.3 (360.5–562.2)	244.4 (142.8–346.0)	0.0011	461.3 (360.5–562.2)	289.3 (147.8–430.8)	0.0079	1.5 (1.3–2.0)	3.0 (2.0–5.8)	0.0038
Women (*n* = 12)	233.0 (175.9–290.1)	58.26 (44.72–71.80)	<0.0001	637.8 (482.6–792.9)	277.0 (207.4–346.5)	0.0002	645.4 (477.7–813.2)	322.4 (231.4–413.4)	0.0019	1.4 (1.0–1.5)	2.0 (2.0–4.5)	0.0534

**Table 3:** Ratios of geometric means for the pharmacokinetic parameters after a single oral dose of 100 mg BCP. BCP was administered either as self-emulsifying drug delivery system (BCP SEDDS) or as BCP neat oil (geometric means ± 90% CI).

Subjects	Pharmacokinetic Parameters								
	C_max_ [ng/mL]			AUC_0–12h_ [ng/mL × h]			AUC_0–24h_ [ng/mL × h]		
	Geometric Means		Ratio of Geometric Means (90% CI)	Geometric Means		Ratio of Geometric Means (90% CI)	Geometric Means		Ratio of Geometric Means (90% CI)
	BCP- SEDDS	BCP Neat Oil		BCP- SEDDS	BCP Neat Oil		BCP- SEDDS	BCP Neat Oil	
All (*n* = 24)	186.7	51.2	3.6 (2.92–4.55)	510.3	227.0	2.2 (1.85–2.73)	511.8	253.3	2.0 (1.61–2.53)
Men (*n* = 12)	161.9	48.1	3.4 (2.31–4.91)	437.0	203.5	2.1 (1.56–2.96)	437.0	223.9	2.0 (1.34–2.84)
Women (*n* = 12)	215.3	54.46	4.0 (3.00–5.22)	595.9	253.2	2.4 (1.82–3.04)	599.5	286.6	2.1 (1.54–2.84)

## Data Availability

The data presented in this study are available on request from the corresponding author. The data are not publicly available due to patent/privacy reasons.

## Supplementary Materials

The following supporting information can be downloaded at: https://www.mdpi.com/article/10.3390/molecules27092860/s1: Figure S1: Chromatogram of standard Caryophyllene (β = 40 ng/mL) and an example plasma sample of a subject. Table S1: Technical information regarding BCP sample analysis using the GC-MS/MS technique.

## Sample Availability

Not available.
