# Supplementary material for: Enhanced Oral Bioavailability of β-Caryophyllene in Healthy Subjects Using the VESIsorb® Formulation Technology, a Novel Self-Emulsifying Drug Delivery System (SEDDS)
Source: Molecules. 2022 Apr 30;27(9):2860. doi: 10.3390/molecules27092860 (PMC9104399; doi:10.3390/molecules27092860)

Supplementary **Table S1:** Settings of the final EI – GC-MS/MS method operated in MRM mode.

Analyte	Precursor ion (m/z)	Product ion (m/z)	Dwell time [ms]	Cone voltage [V]
β-Caryophyllene (BCP)	204	93	40	10
		147 (Q)		5
		121		5
IS (Humulene)	204	93	40	10
		147 (Q)		5
		133		5

MRM: multiple reaction monitoring, Q: Quantifier ion transition.

GC-MS/MS system details: ZB-MR1ms column (dimension: 30 m x 0.25 mm x 0.25 µm; Art: 7HG-G016-11), splitless injection with 2 µL at 250 °C with 1.0-1.2 mL/min column flow and 3 mL/min purge flow;

GC-parameter: collision gas (N_2) at 1.5 mL/min and quench gas (He) at 2.25 mL/min (MS/MS 7000 C) or 4.0 mL/min (MS/MS 7010 B) at constant flow and 280 °C transfer line temperature; Run time: appr. 10 min.

Oven program:

Heating rate [°C/min]	Temperature [°C]	Hold time [min]
initial	40	0.5
40	150	0.0
15	185	0.0
60	320	2.0

MS-Parameter: Ion Source EI, 230 °C source temperature, fixed electron energy (70 eV), 4 min solvent delay; 200 – 400 V relative EMV Delta.

Supplementary Figure S1: Chromatogram of standard Caryophyllene (β = 40 ng/mL) and an example plasma sample of a subject.

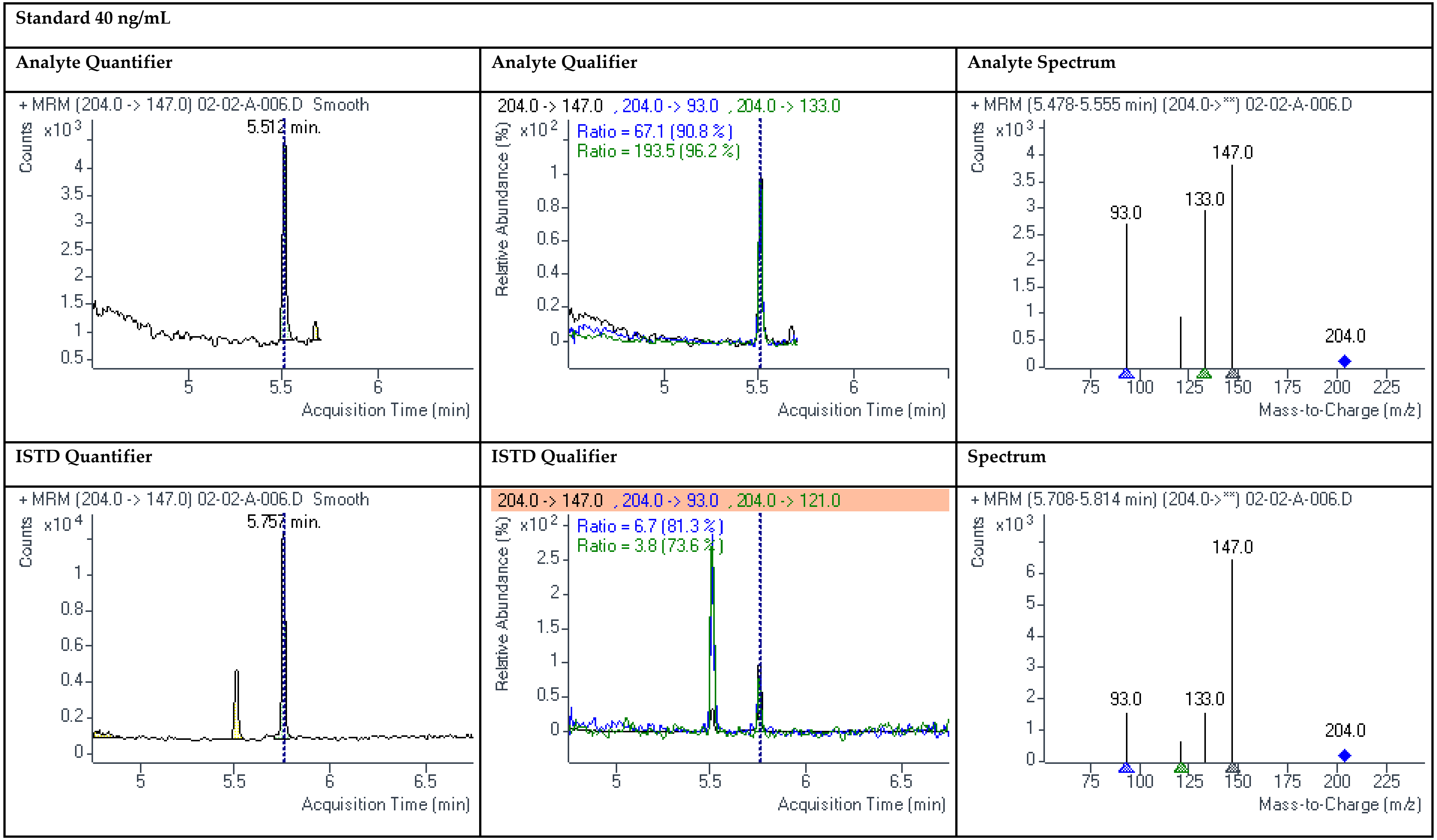

Example plasma sample

Analyte Quantifier

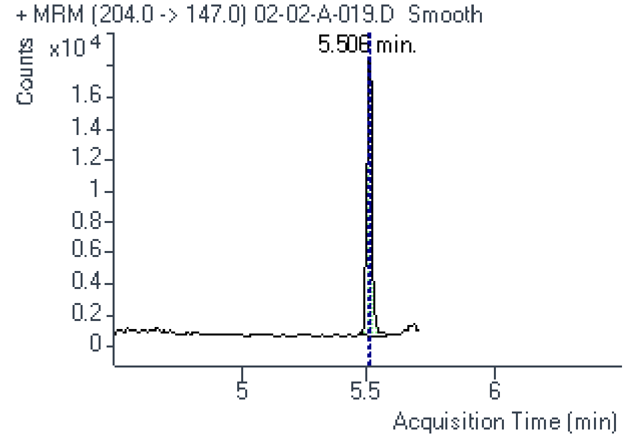

Analyte Qualifier

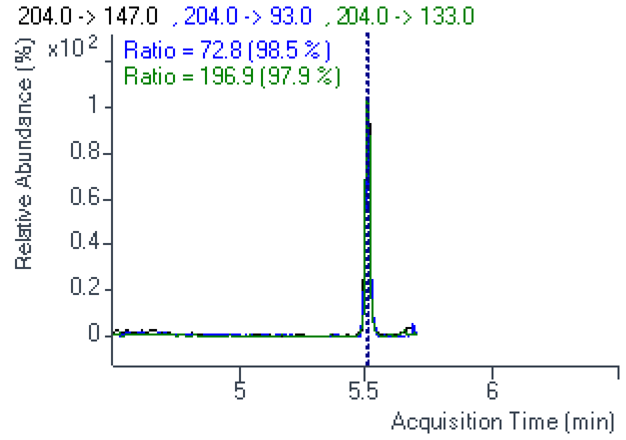

Analyte Spectrum

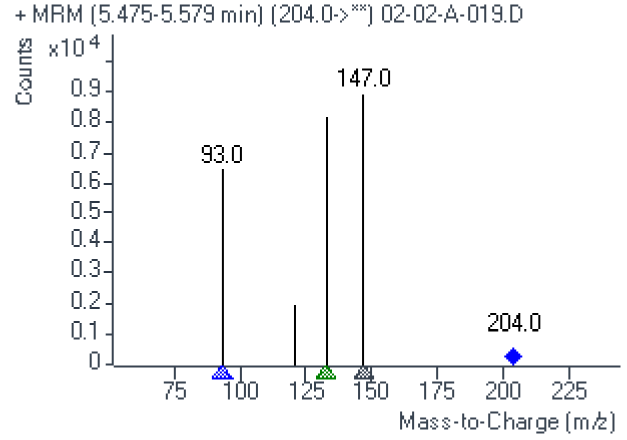

ISTD Quantifier

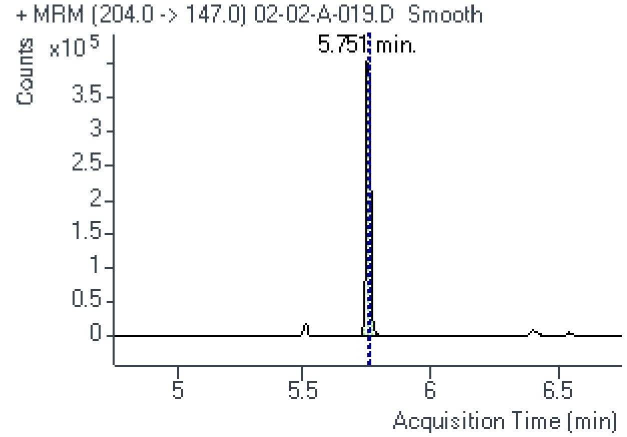

ISTD Qualifier

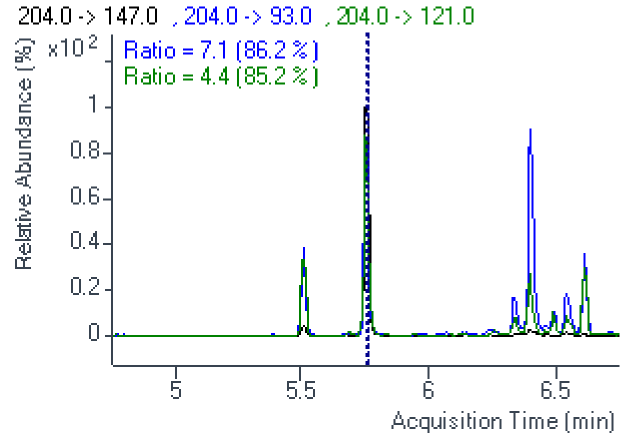

Spectrum

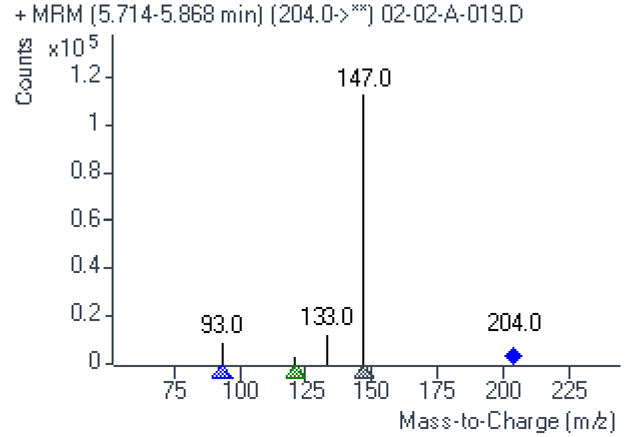

Supplement: Supplementary file 1 [file molecules-27-02860-s001.zip › molecules-1696791-supplementary.pdf]
